# Self-transcendence accompanies aesthetic chills

**DOI:** 10.1371/journal.pmen.0000125

**Published:** 2024-10-04

**Authors:** Leonardo Christov-Moore, Felix Schoeller, Caitlin Lynch, Matthew Sacchet, Nicco Reggente

**Affiliations:** 1 Institute for Advanced Consciousness Studies, Santa Monica, California, United States of America; 2 Consciousness Center of Oaxaca, Oaxaca, Mexico; 3 Meditation Research Program, Department of Psychiatry, Massachusetts General Hospital, Harvard Medical School, Boston, Massachusetts, United States of America; University of Sousse Faculty of Medicine of Sousse: Universite de Sousse Faculte de Medecine de Sousse, TUNISIA

## Abstract

Self-transcendence (ST) is a state of consciousness associated with feelings of ego-dissolution, connectedness, and moral elevation, which mediates well-being, meaning-making, and prosociality. Conventional paths to ST, like religious practice, meditation, and psychedelics, pose nontrivial barriers to entry, limiting ST’s study and application. Aesthetic chills (henceforth “chills”) are a psychophysiological response characterized by a pleasurable, cold sensation, with subjective qualities and downstream effects similar to ST. However, evidence is lacking directly relating chills and ST. In the summer of 2023, we exposed a diverse sample of 2937 participants in Southern California to chills-eliciting stimuli, then assayed chills, mood and ST. Even after controlling for differences in demographics, traits, and prior affective state, both chills likelihood and intensity were positively associated with measures ST. Parametric and non-parametric analyses of variance, mutual information, and correlation structure found that chills occurrence and intensity, and ST measures are reliably interrelated across a variety of audiovisual stimuli. These findings suggest aesthetic chills may denote sufficiently intense feelings of self-transcendence. Further study is necessary to demonstrate the generalizability of these results to non-WEIRD populations, and the precise direction of causal relationships between self-transcendent feelings and aesthetic chills.

## Introduction

Self-transcendence (ST) is a positive, transformative state of consciousness [1] characterized by elevated mood, ego-dissolution (a perceived dissolution or blurring of the boundaries of self), connectedness (a felt connection to self, others, world, and environment), and moral elevation (a motivation to live in a noble, virtuous way), consistent with accounts of mystical experience [2, 3]. The original theory of ST is based in clinical nursing and was originally derived from inquiry on well-being in older adults [1, 4], where it was defined as the capacity to transcend one’s personal needs and desires; connect with others, the environment, and a spiritual dimension or God; and to extend concern to others. ST may encourage and accompany human flourishing, being associated with a sense of coherence, purpose and resilience [5], as well as self-esteem, hope for the future, and emotional well-being [1, 4, 6].

Across many populations, individuals with high self-transcendence scores tend to have better mental health and well being [7–11], presenting lower levels of depressive symptoms [12], and higher levels of self-esteem and internal locus of control [13, 14]. These relationships are consistent across many studies, according to a recent meta analysis [15]. Beyond day-to-day wellbeing, ST may mitigate suffering and enhance personal meaning in the context of difficult, life-changing events [6, 16]. ST has been cited as a positive mediator of resilience and well-being in caregivers of the elderly with dementia [16], and in the resolution of grief following personal loss and death [17]. Nursing staffs’ self-transcendence was related to their level of sustained caring and well-being [18]. ST has been shown to mediate transitions from disequilibrium to well-being in cancer patients [1]. ST may similarly aid the alleviation of suffering during times of radical uncertainty, such as during the recent COVID-19 pandemic [19]. ST is also implicated as a mediator of increased positive disposition towards others, as following transformative experiences such as awe, i.e. challenge exceeding the scope of one’s mental structures [20, 21], immersion in nature [22], loving kindness and mindfulness meditations [23]; or in the case of community-oriented public policy interventions [24], social activism [25], and environmental activism [26].

Mechanistic accounts view ST as having aspects both interpersonal, relating to self, and intrapersonal, relating to others [12]; functioning both as a means to integrate all dimensions of the self, as well as a reach beyond self-boundaries [4]. This is thought to occur via two main pathways: first, ST can turn rigid, defensive self-focus into flexible and receptive self-construal. Second, ST can increase positive other-focus by integrating reward and other-oriented social signals in the brain, possibly via control and mentalizing processes used in inferring the needs, desires and feelings of others [18]. ST’s reshaping of reward systems may hence have implications for chronic pain and substance abuse disorders [27]. Research and theory relevant to ST’s transformative effects propose that belief structures are predicated on demarcated internal/external states [28], and hence that dissolving the partition separating these via the ego-dissolving aspects of ST [29] may allow for otherwise impossible cognitive/behavioral restructuring towards prosocial ends [30]. Indeed, ST is increasingly thought to mediate the prosocial changes in values resulting from psychedelic experience [31] through a process known as “unselfing”, that is, a deep persistent turning of attention away from the self on to the world. Our group has enhanced prosociality by both a) disruptive neuromodulation of cortical systems involved in self-other distinction systems [32, 33] and b) having participants virtually exchange their bodies using virtual reality [34]. These findings suggest that, across a wide variety of experiences and practices, a felt dissolution of self-boundaries, coupled with a sense of connectedness with something beyond one’s self, appears to be a central mechanism in shifting belief and behavior toward prosociality.

While major life transitions can trigger ST [35], traditional approaches to ST include mystical/spiritual practices, advanced meditative practice, and psychedelic substances. ST mediates the positive outcomes and spiritual well-being in the Amish [36], and within Christian healthcare missions [16]. Recent studies examining ST and personality in relation to mindfulness found significant but modest relationships between ST and neuroticism, openness to experience, extraversion, and agreeableness [23, 37]. Persistent ST has been shown to arise from meditation programs [38]. ST has been found to mediate the efficacy of MDMA-assisted therapy in addressing social anxiety disorder [39], and studies of immediate and long-term effects of psilocybin have shown profound increases (effect sizes on the order of Cohen’s d = 1.9–2.7) in felt internal and external unity, and transcendence of time and space [2, 40]. Despite the utility of spirituality, meditation, and psychedelic substances for evoking ST, they are neither universally nor easily accessible. Meditation can require many hours of dedicated practice, psychedelics have numerous pharmacological, cultural, and legal contraindications, and spiritual practices’ “packaging” can create resistance and limit universality in secular or multi-denominational populations. In addition, investigators have noted a lack of clarity in operationalizing ST and a preponderance of approaches that are complex, obscure and “imprecisely spiritual” [41], impeding its wide adoption. In this context, further research is necessary to democratize and facilitate ST as a resource for all populations.

Strong aesthetic experiences may serve as one such replicable and accessible path to ST. Aesthetic chills are a pleasurable psychophysiological response (not to be confused with chills arising from horror or cold) that denote such strong responses to audio and/or visual aesthetic stimuli, characterized by a cold sensation, shivering, and goosebumps, often along the spine [42–45]. Aesthetic chills (henceforth”chills”) have been reported in response to a wide variety of content, including art (music, literature, poetry), science (lectures, documentaries), and religious content (choral pieces, ritual) [43, 46]. Individuals vary substantially in their susceptibility to chills and the intensity with which they experience them, with recent studies relating the likelihood of experiencing chills to openness to experience, absorption, extroversion, and positive arousal [42–46]. Importantly, chills seem to enhance affect, awe, meaning, and prosociality [47–50], outcomes consistent with psychedelic, mystical, or ST experiences. In summary, circumstantial evidence suggests that aesthetic chills may be a marker of aesthetically-evoked ST that is: (1) replicable [43, 44], (2) correlated with recognizable physical markers such as tingling sensations, piloerection (goosebumps), and shivering [51, 52], and (3) linked to neurophysiological markers [42].

Here we sought to examine whether whether strong aesthetic responses to audiovisual stimuli show the transformative, prosocial subjective characteristics typically associated with psychedelics and advanced meditative states. We hypothesized that participants who report aesthetic chills would also report higher levels of self-transcendence (ego-dissolution, interconnectedness, and moral elevation) than those who did not report chills. Furthermore, we hypothesized that the intensity of chills among those who reported chills would be correlated with these three facets of self-transcendence, even after controlling for trait differences, change in mood, and stimulus.

## Materials and methods

### Participants

3,259 participants were recruited and initially took part in the experiment between April 11th and August 3rd of 2023. Following quality assurance procedures (details in Materials and Methods: Quality assurance and assumption testing), analyses were conducted on a diverse group of 2,937 participants, all residents of Southern California. The gender distribution was fairly balanced, with 54.24% identifying as female, and 41.44% as male. In terms of political affiliation, the largest group identified as Democrats (50.66%), followed by Republicans (21.59%), and Independents (14.81%). 11.64% of participants did not specify a political affiliation, while a small proportion (1.19%) identified with other political affiliations. The majority of participants (68.44%) identified as White or Caucasian followed by those identifying as Other (11.37%), then Indian/Native American or Alaska Native (4.97%), Black or African American (1.46%), and Asian (0.31%).

### Procedure

Participants were recruited for this study through an online platform (Qualtrics.com) with a focus on individuals residing in Southern California. Before proceeding with the study, participants underwent an initial screening to confirm their geographical location and provided their informed consent. Participants were then asked to provide basic demographic information including gender, education level, and age. Additionally, participants were queried about their political orientation and whether they were affiliated with any political party. In order to assess participants’ emotional state, they were prompted using visual likert scales to indicate their levels of arousal (1 = calm to 7 = agitated/excited) and valence (1 = unpleasant to 7 = pleasant), as well as their mood (1 = sad/angry to 7 = happy). Subsequently, participants completed several questionnaires to assess trait dispositions, including the Dispositional Positive Emotion Scale (DPES) [53], NEO Five-Factor Inventory (NEO-FFI-3) [54], Modified Tellegen Absorption Scale (MODTAS) [55], and Kama Muta Frequency Scale (KAMF) [56]. These scales are described in detail in Trait Measures, below. Participants were then randomly assigned to one of 40 video stimuli. After exposure to the assigned stimulus, participants were asked to report their emotional state in terms of valence and arousal once again. They were also asked to indicate their level of liking for the video, whether they had seen the video previously (yes/no), whether they experienced chills while watching the video (yes/no), and if so, to rate the frequency (0-more than 2) and intensity of their chills (0–100). Participants were also asked whether the video reminded them of a personal experience (yes/no), and if they experienced goosebumps (yes/no) or tears (yes/no), they were asked to indicate what elicited those responses (open form text). Following the assessment of participants’ immediate responses to the stimulus, they were directed to complete a set of state questionnaires including the Watts Connectedness Scale (WCS) [57], Kama Muta Frequency Scale (KAMF) [56], and Ego-Dissolution Inventory (EDI) [58]. Upon completion of the study, participants were thanked for their participation and remunerated via cash and/or gift cards via the Qualtrics platform in the amount of $12/hour, with total time rounded up to the nearest 15 minutes. The average duration of each experiment was approximately 37 minutes.

### Stimuli

We selected 40 stimuli (20 audio and 20 audiovisual) combining a subset (N = 10) from the original ChillsDB [44] and a novel subset obtained from additional parsing using the ChillsDB method and internal polling [43]. Each of the 20 stimuli was presented to participants in either two formats ‐ audiovisual or audio-only ‐ in order to compare and test for differences between the two presentation modalities. The ChillsDB is an open-source database of validated audiovisual stimuli eliciting aesthetic chills (goosebumps, psychogenic shivers) in a population based in the United States. The database consists of 204 chills-eliciting videos in three categories: music, film, and speech. ChillsDB was built using an ecologically-valid method for finding chills stimuli “in the wild” by searching for mentions of somatic markers in user comments using algorithms to parse social media platforms (YouTube and Reddit). An updated ChillsDB (2.0) including the stimuli with accompanying demographic, trait and state measures reported here, is now available [43].

### Trait measures

The Dispositional Positive Emotion Scale (DPES): The DPES [53] measures one’s dispositional tendencies to feel positive emotions towards others in their daily lives. The DPES consists of seven subscales, which measure ones temperament regarding joy, contentment, pride, love, compassion, amusement, and awe. The scale comprises a total of 38 items, with each subscale containing 5 or 6 items. Participants rate their agreement with statements such as”On a typical day, many events make me happy” on a 7-point Likert scale ranging from”1 ‐ strongly disagree” to”7 ‐ strongly agree.” The total score is obtained by averaging the item responses, yielding a range from 1 to 7, with higher scores indicating greater levels of positive emotion. NEO Five-Factor Inventory (NEO-FFI-3): The NEO-FFI-3 [54 is a widely used personality assessment tool based on the Five-Factor Model (FFM) of personality. It is designed to measure five broad dimensions of personality: neuroticism, extraversion, openness to experience, agreeableness, and conscientiousness. The NEO-FFI-3 consists of 60 items, with 12 items for each of the five personality factors. Participants respond to statements such as”I worry a lot” or”I am talkative” on a 5-point Likert scale, indicating the extent to which each statement reflects their own personality traits (1 = not at all, to 5 = very much). The NEO-FFI-3 provides researchers and practitioners with a concise and reliable measure of the key dimensions of personality, allowing for a comprehensive understanding of individuals’ typical patterns of behavior, emotions, and cognition. The constituent items of NEO-FFI include negative affect, self-reproach, positive affect, sociability, activity, aesthetic interests, intellectual interests, unconventionality, nonantagonistic orientation, prosocial orientation, orderliness, goal-striving, and dependability. Modified Tellegen Absorption Scale (MODTAS): The Tellegen Absorption Scale (TAS) is a 34-item multi-dimensional measure that assesses imaginative involvement and the tendency to become mentally absorbed in everyday activities [59]. We employed a modified version, the MODTAS, which has a Likert scaled response format and a clearer covariance structure than the original TAS [55]. Subjects are asked to rate the frequency of their experiences on a 5 point scale, ranging from 0 (never) to 4 (very often). It consists of 5 correlated primary factors: Synaesthesia, Altered States of Consciousness, Aesthetic Involvement in Nature, Imaginative Involvement and apparent experiences of Extra Sensory Perception. Kama Muta Frequency Scale (KAMF) : This 7-item scale [56] measures predisposition for Kama muta, (meaning: ‘moved by love’) an affective state described as ‘being moved’, ‘heart-warming’, ‘stirring’, or ‘being emotionally touched’. Kama muta is a distinct positive social relational emotion, evoked by experiencing or observing a sudden intensification of communal sharing. It motivates affective devotion and commitment to community sharing. The construct has been validated with studies in 15 languages, over 19 countries, across 5 continents. It is commonly accompanied by a feeling of warmth in the chest, tears or moist eyes, chills or piloerection, feeling choked up, buoyancy, and exhilaration.

### State outcome measures

Chills Likelihood: Participants were asked to report whether they experience chills (Yes/No). Chills Intensity: Participants were asked to report the intensity of their chills from 0–100 following the single video stimulus. Following this, they completed three measures of the principal components of Self-Transcendence. Ego-Dissolution Inventory (EDI): The EDI [58] consists of sixteen items relating to altered ego-consciousness, eight relating to the experience of ego-dissolution (comprising the EDI), and eight relating to the antithetical experience of increased self-assuredness, termed ego-inflation. Items are rated using a visual analog scale ranging from 0 to 100. Watts Connectedness Scale (WCS): The WCS is a new three-dimensional index of felt connectedness that may sensitively measure therapeutically relevant psychological changes post-psychedelic use [57]. Items cluster into: connectedness to self (e.g.”My mind felt connected to my heart/emotion.”), connectedness to others (e.g.“I felt connected to friends and/or family.”), and connectedness to the wider world and spirituality (e.g.”I felt that everything is interconnected.”). State Moral Elevation Scale (SMES): The SMES [60] assays positive feelings following the observation of another performing a prosocial act, it consists of 9 items assessing, on a Likert-type 5-point scale, the extent to which the participant had different experience, measuring Emotional Reaction (“in touch with the better parts of myself”), Physical Reaction (“a warm or glowing feeling in my chest”), and Motivation (“motivated to live in a nobler or virtuous way”). While not all stimuli used here had explicitly prosocial acts in them, the wording of this question (“to what extent did you experience each of the following statements while watching the video?”) did not preclude non-explicitly prosocial stimuli and was appropriate to gauge general moral elevation following the specific content of each stimulus.

### Ethics statement

The experiment is in compliance with the Declaration of Helsinki. Following review, the study protocol was granted an exemption status by Advarra IRB (Pro00068209). We obtained voluntary written informed consent from all participants in conformity with the Ethics Code of the American Psychological Association. Thus, all participants were informed about the purpose of the research, their right to decline to participate and to withdraw from the experiment, and the limits of confidentiality. We also provided them with a contact for any questions concerning the research and with the opportunity to ask any questions regarding the phenomenon under study (aesthetic chills) and receive appropriate answers.

### Analyses

#### Analysis software

Mutual information analysis was conducted in python, using the sklearn module. All remaining analyses were conducted in R using the anovaNP, seolmatrix, and descriptives packages implemented within Jamovi [61–63].

#### Quality assurance and assumption testing

Preliminary analysis revealed that 932 participants reported a non-zero chills intensity (Mean I = 19.6, SD = 25) despite reporting that they did not experience chills. This was further corroborated in their qualitative descriptions, where the majority stated explicitly they did not experience chills. We eliminated all instances (N = 219) where chills intensity exceeded 1 standard deviation from the mean (Mean I = 10.3, SD = 20.7). We retained only those participants (N = 656) in whose qualitative responses unambiguously confirmed the absence of chills, these participants were placed in the ‘No Chills’ category, and subsequently omitted from the Chills-only analysis. In order to choose appropriate analyses, we examined the normality of the data using visual inspection of histograms and Q-Q plots. Although analyses of measurements in excess of 30 data points can often be treated as approximately normal due to the central limit theorem [64], we wished to ensure Q-Q plots confirmed that assumption. Trait measures were approximately normal for both the full cohort and chills-only cohorts (S1 and S2 Figs) and showed expected factor loading (S2 Table). State outcome measures for the full cohort showed high “zero loading” due to the large amount of zero and near-zero responses. Thus, we opted for non-parametric ANOVA (Kruskal-Wallis) to compare outcomes between chills and non-chills responders, and mutual information to examine directional relationships between traits and outcomes in the full cohort. Outcome measures for the chills-only cohort appeared approximately normal (S1 and S2 Figs) and suitable for parametric tests ‐ thus we employed partial and full correlations to examine relationships between demographics, traits, chills intensity and ST measures. To examine whether the relationship between ST and chills likelihood/intensity differed significantly across stimuli, we performed a binomial regression of ST measures (ego-dissolution, connectedness, and moral elevation) against chills likelihood, and a linear regression of ST measures against chills intensity, and in both included interaction terms (e.g. ego-dissolution*stimulus).

#### Binary chills response and mutual information analyses

To accommodate non-normality in the full cohort, we performed a Kruskal-Wallis non-parametric ANOVA comparing outcome measures between participants who reported chills and those who did not. To examine non-parametric, directional relationships within and between traits, demographics and outcomes, we employed a mutual information analysis, as this approach can best accommodate non-normality, discrete variables, and non-linear relationships, while also indicating directionality. As each analysis quantifies the reduction in uncertainty in a variable Y created by measuring a variable X, the output may vary when analyzing Y with regard to X. To assess the threshold for significance, we simulated a null distribution by randomizing values within each variable columnwise and repeating the pairwise analyses 5000 times. The threshold for significance at = .05 was set by the 95th percentile value for the bootstrapped null distribution. Thresholds varied across variables, between .013 and .029. We opted for a general threshold of .03 in order to focus the discussion on the most significant results.

#### Chills intensity analyses

Given the approximate normality in outcome measures within the chills-only cohort, we proceeded with parametric analyses. We performed a correlation analysis between all outcomes, and then a partial correlation analysis controlling for stimulus, trait and demographic variables, to assess the relationship between ST and chills intensity. We also examined the contribution of trait and demographic factors to this relationship by comparing correlation values, achieved by transforming the correlation coefficient values into z scores using Fisher’s r to z transformation, so that the z scores could be compared and analyzed for statistical significance. We accompanied these analyses with graphical gaussian plots of correlation structure to more easily visualize these relationships, using the seolmatrix R package [62] implemented in Jamovi. Additionally, in order to examine further whether chills intensity correlate with one”self-transcendence” factor represented by ego-dissolution, connectedness, and moral elevation, or whether it has unique, nonoverlapping relations with each of these three constructs, we performed a k-means cluster analysis using the snowcluster R package [63], which included assessing the optimal number of clusters, and a principal components analysis (PCA) to assess principal axes along which clusters were distinguished. We additionally examined the correlation between chills intensity and the principal eigenvalue from a PCA of the ST facets, and a linear regression of each ST facet against chills intensity.

#### Data availability statement

The full ChillsDB stimuli database [43, 44] is available in the figshare repository, as part of this record, DOI:10.6084/m9.figshare.23935611.v1. The dataset generated by the survey research is released under a Creative Commons Attribution 4.0 International (CC BY 4.0) license on FigShare https://doi.org/10.6084/m9.figshare.23935611.v1. The dataset is divided into two .csv files available under a CC BY 4.0 license on the associated FigShare (ChillsDB2). For a comprehensive understanding of each column, researchers are advised to refer to the Header Explanation File. All code supporting these analytical efforts is included in the following repository. Note: Requires the LibSVM toolbox. https://github.com/Institute-for-Advanced-Consciousness/E4-F01.

## Results

Broadly, we took an approach similar to that employed in memory research paradigms [53]. Rather than employ an explicit control condition as independent variable, all participants were exposed to the same stimuli, and we examined the trait predictors and (a) reported characteristics of those exposures resulting in our outcome measure of interest (in this case chills rather than recall), as well as (b) the intensity of reported chills in these exposures. Binomial and linear regression analyses examining interactions between stimulus identity and ST measures showed no significant ST*stimulus interaction effects, indicating that the relationship between ST measures and chills did not vary significantly across stimuli.

### Differences between chills responders and non-responders

#### Non-parametric comparisons

**Fig 1:**
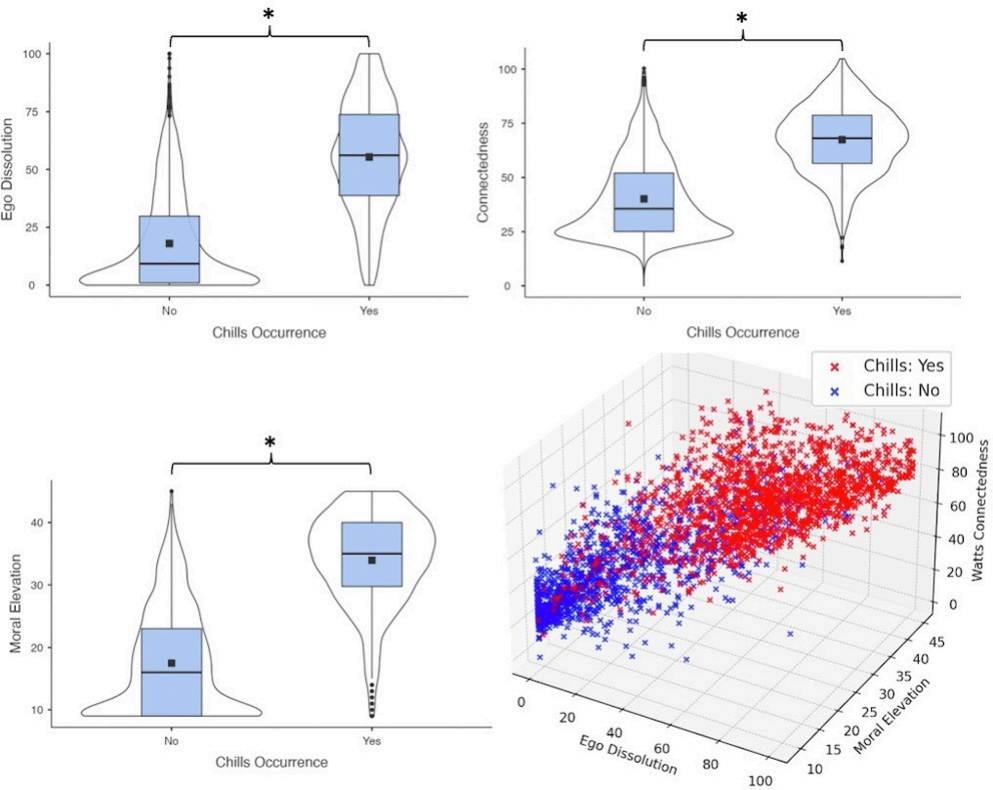
Differences in self-transcendence measures between participants who reported experiencing chills and those who did not.

Chills responders were designated as those who responded “Yes” to the Chills Likelihood question (“Did you experience chills?”). A Kruskal-Wallis non-parametric ANOVA found significant differences in all outcome measures between chills and non-chills responders (see 1), though coefficients and effect sizes were 2 orders of magnitude higher for ST measures when compared with affective measures (see Table 1). Participants reporting chills reported significantly higher Ego Dissolution (χ2 = 1221.4, p < .001, ε2 = .4184), Connectedness (χ2 = 1191.7, p < .001, ε2 = .4059), and Moral Elevation (χ2 = 1516.2, p < .001, ε2 = .5164). These differences are illustrated in Fig 1. They also reported significantly larger increases, from pre- to post-stimulus, in arousal (χ2 = 12.8, p < .001, ε2 = .0044), valence (χ2 = 34.9, p < .001, ε2 = .0119), and mood (χ2 = 28.7, p < .001, ε2 = .0098).

**Table 1:** Descriptive statistics and Kruskal-Wallis non-parametric ANOVA of outcome measures outcome measures for chills responders (n = 1507) and non-responders (n = 1430).

Chills Y/N	Chills Intensity	Ego Dissolution	Connectedness	Moral Elevation	ΔArousal	ΔValence	ΔMood
Mean No	0	18	40.1	17.5	0.161	0.041	0
Yes	63.9	55.4	67.4	34	0.499	0.657	0.183
Median No	0	9.25	35.6	16	0	0	0
Yes	66	56.1	68.1	35	0	0	0
SD No	0	20.8	17.4	8.35	2.95	2.98	1.06
Yes	25.2	23.9	15.7	7.36	3.26	2.76	1.11
*χ* ^*2*^	2466	1221	1192	1516	12.8	34.9	28.7
*p*	< .001	< .001	< .001	< .001	< .001	< .001	< .001
*ε* ^*2*^	0.84	0.418	0.406	0.516	0.004	0.012	0.009

#### Mutual information analysis

**Fig 2:**
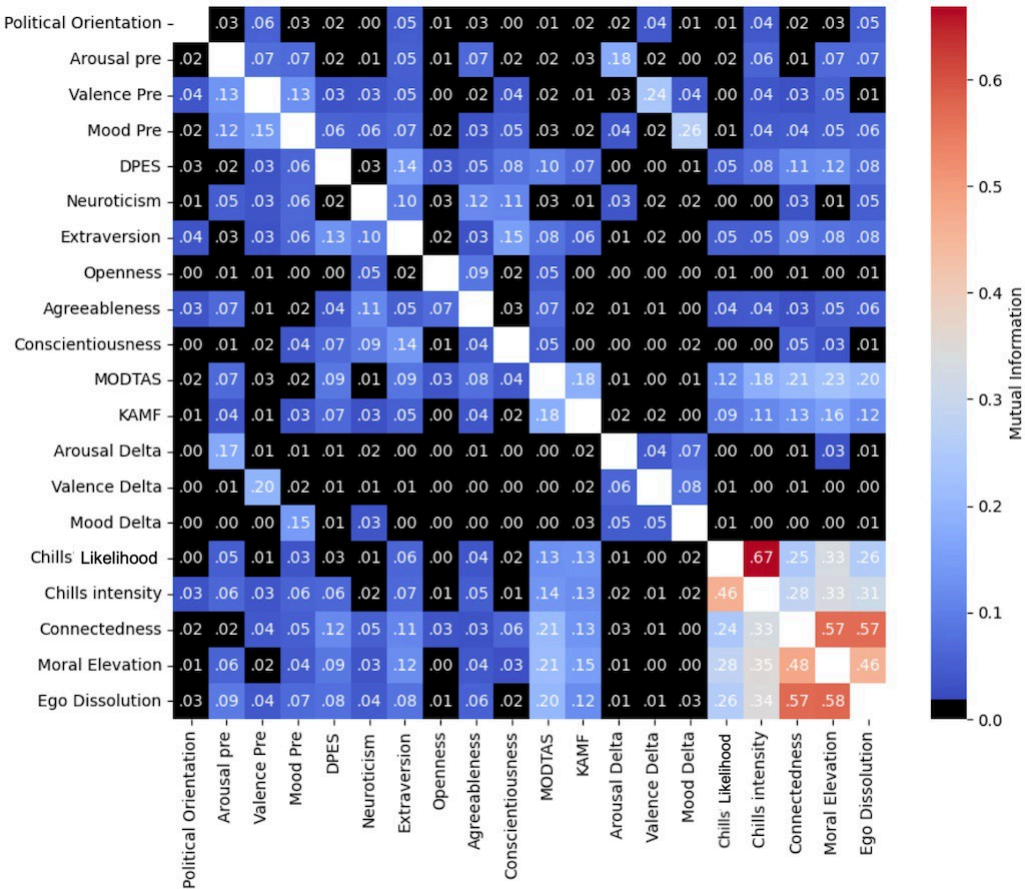
Mutual information in full cohort between traits, demographic variables, and outcomes. Cells in black fall below the bootstrapped general threshold (.03) for significance at p < .05. Cell values are rounded to 2 decimal places. Coefficients indicate the extent to which measurement of X (row variable) reduces uncertainty about Y (column variable).

We non-parametrically examined mutual dependency between demographic, trait, and outcome measures using a mutual information (MI) analysis, quantifying the amount of information obtained by each variable by observing the other. These show approximately four-fold greater MI between chills likelihood/intensity and ST measures, than that for any other relationship with MI above the bootstrapped = .05 threshold (See Fig 2). For a deeper inquiry into the contribution of demographics and trait variables to chills likelihood and intensity, see [65].

### Relationships with chills intensity

#### Correlations and partial correlations

When comparing correlation coefficients before (S1 Table) and after controlling for stimulus, demographic and trait variables(see Table 2), the following significant differences emerged: Correlation coefficients (r) diminished between Chills Intensity and Ego Dissolution (.516 to .389, p < .0001), Connectedness (.48 to .397, p = .004), and Moral Elevation (.506 to .356, p < .0001); between Ego Dissolution and Moral Elevation (.55 to .446, p = .0001); and between Connectedness and Moral Elevation (.643 to .553, p = .0001).

**Table 2:** Partial correlations between outcome measures controlling for trait and demographic measures, stimulus, pre-stimulus affective state, and prior exposure.

	Chills Intensity	Ego Dissolution	Connectedness	Moral Elevation	ΔArousal	ΔValence
Chills Intensity						
Ego Dissolution	.389*	.	.	.	.	.
Connectedness	.397*	.490*	.	.	.	.
Moral Elevation	.356*	.446*	.553*	.	.	.
ΔArousal	.021	.007	-.049	.006	.	.
ΔValence	.062	.033	.100	.078	.018	.
ΔMood	.067	.052	.100*	.055	-.081*	.243*

#### Correlation structure

**Fig 3:**
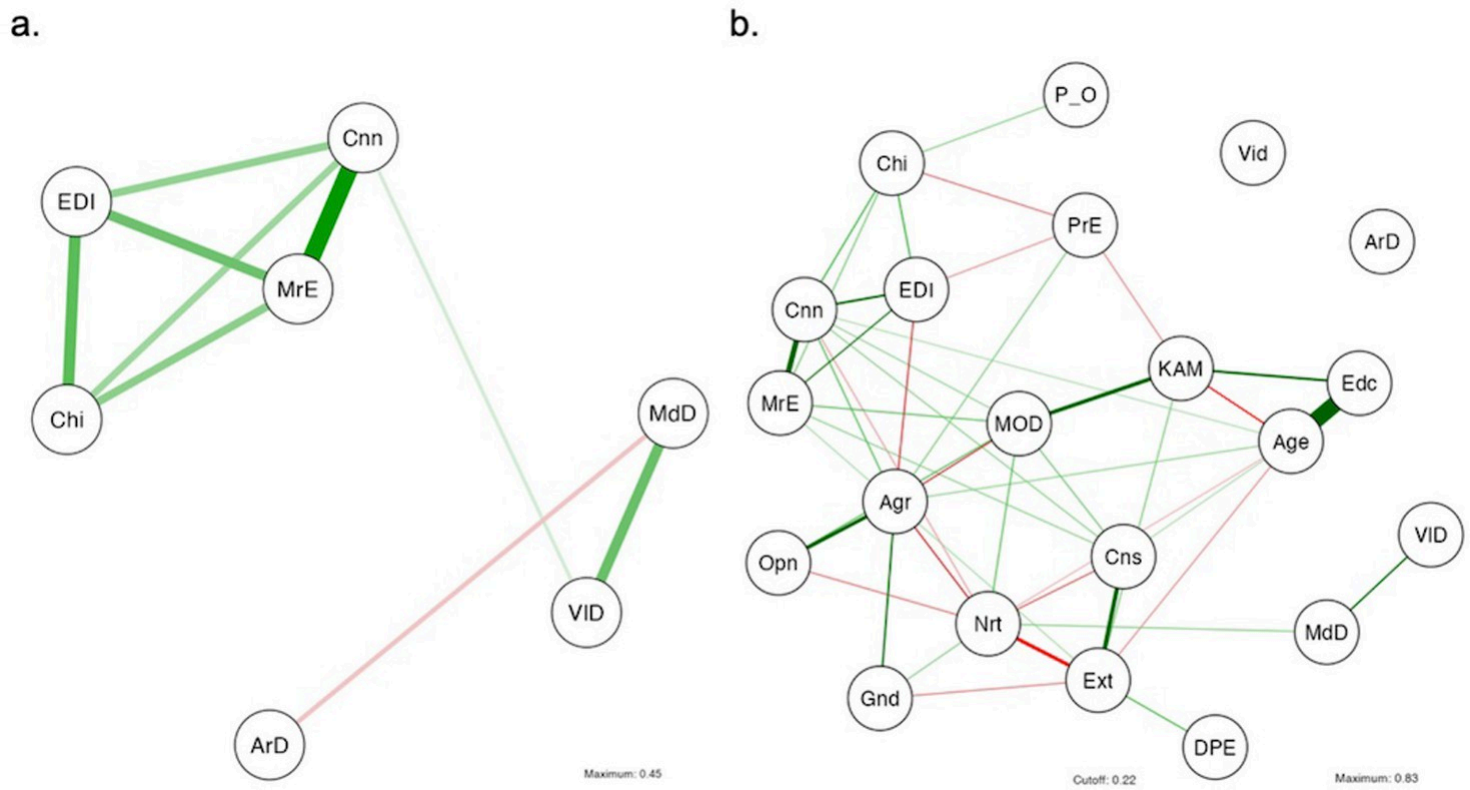
Correlation structure in chills-only participants between (a) outcomes only and (b) Outcomes and traits/demographics. Chi = chills intensity, EDI = ego dissolution, Cnn = connectedness, MrE = moral elevation, MdD = mood delta, VlD = valence delta, ArD = arousal delta, PO = political orientation, PrE = prior exposure, Vid = video, MOD = absorption, KAM = kamamuta, DPE = positive emotionality, Agr = agreeableness, Opn = openness, Nrt = neuroticism, Cns = conscientiousness, Ext = extroversion, Gnd = gender, Edc = education.

The correlation structure of the correlation and partial correlations is illustrated in Fig 3. In either case, chills intensity, ego dissolution, connectedness, and moral elevation form a tightly interrelated construct.

#### Cluster and principal components analysis

**Fig 4:**
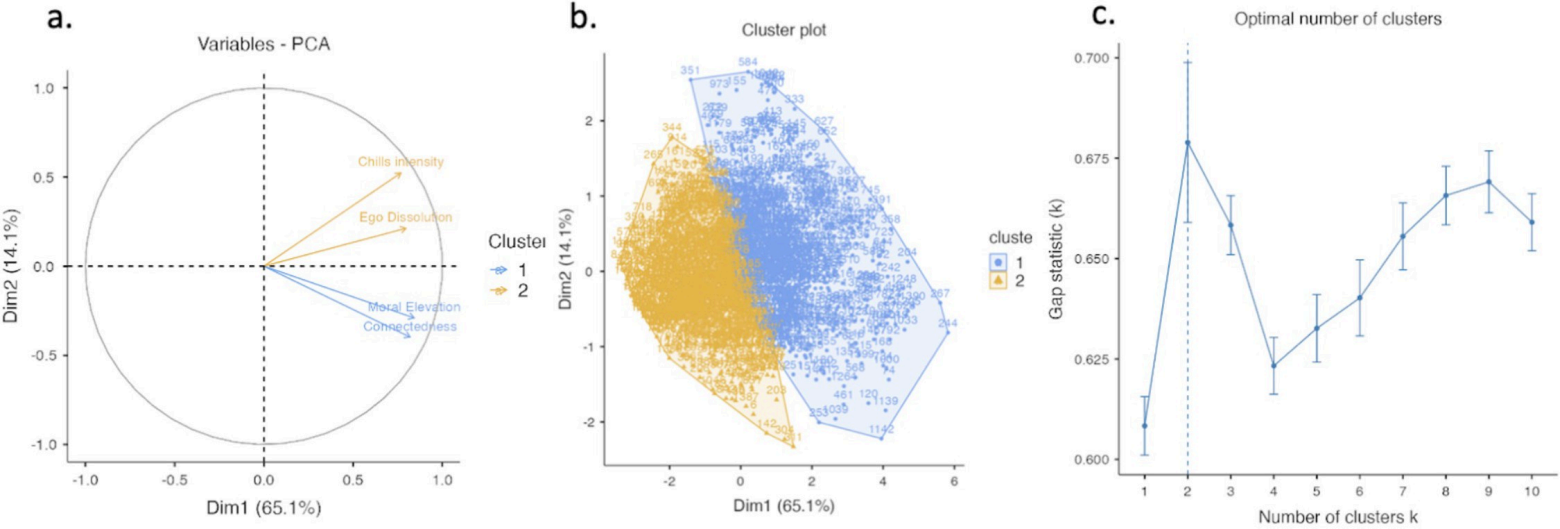
Cluster analysis of ST and chills intensity provides evidence of a covariation along roughly two principal components. (a) principal components of variance in ST and chills intensity (b) clusters of data along these two components. (c) maximizing for parsimony and gap statistic supports a dual cluster/component model.

Cluster and PCA analysis of ST and chills intensity data showed two cleanly distinguishable clusters along a domain defined by two principal components, chills intensity/ego dissolution, and moral elevation/connectedness (see Fig 4). A PCA of the three ST facets produced a principal”ST” eigenvector accounting for 65% of variance with the following loadings: ego-dissolution = .88, connectedness = .42, moral elevation = .18. This was highly correlated with chills intensity (r = .567). A complementary linear regression of the three ST facets against chills intensity (model fit (r) = .357) showed that they each have significant (p < .001) nonoverlapping contribution to chills intensity, with the following standardized coefficients: connectedness = .184, moral elevation = .225, ego dissolution = .299.

## Discussion

Here we examined whether aesthetic chills arising from chills-eliciting media, both in their occurrence and subsequent intensity, are reliably associated with three aspects of self-transcendence. Parametric and non-parametric analyses support our hypothesis that the onset and intensity of aesthetic chills are strongly related to all three components of ST, that is: ego-dissolution, connectedness, and moral elevation. These results persist whether analyzing the total data set to examine chills likelihood, or solely the subset of participants who experienced chills, to investigate chills intensity alone. The structure of these relationships suggests that pleasurable, aesthetic chills (not to be confused with thermogenic or fear-related chills) are a feature of moderate to intense experiences of ST; that chills intensity and ST are strongly related (rather than chills simply indexing an inflection point in ST but otherwise driven by traits, for example).

Why might self-reports of phenomenology after experiencing chills mirror those of self-transcendent experiences, and how does the aesthetic content that induces chills relate to this sense of ST? Chills are interpreted as a felt accompaniment to the cognitive confirmation of deeply held beliefs [66]. Informational accounts of chills (aesthetic, horror-related, etc.) propose that chills accompany sufficiently deep inferential processes, in which uncertainty/learning related to a deep prior is reduced [66] at higher, more abstract levels of cortical information encoding—a local peak value in a conditional likelihood function of sensory signals given available models throughout the representational hierarchy. Importantly, the local peak value (i.e., modeled as a null derivative) is independent of valence, accounting for the fact that chills can be experienced in response to both positive (appetitive) and negative (aversive) stimuli. The ego-dissolution facilitated by psychedelic ST experiences, for example, has been theorized to be a dissolution of the binding function of a self-model built during early development [29], described as “an ultimately false representation of a simple and enduring substance to which attributes are bound which serves to integrate and unify cognitive processing across levels and domains.” ST is often described as a reconnection with a “truer version of one’s self” [67]. This may be a key factor in facilitating the deep prosocial changes in the self-model occasioned by ST. This idea is beautifully explained by Carl Jung: “Many traditions share versions of a legend in which our journey in life consists of a process of knowing, forgetting, and remembering. We innately know we have come from wholeness, but we are born into a realm that eventually causes us to forget what we once knew, and we spend our lives in search of what will enable us to remember the wholeness we once knew . . .The purpose in having forgotten our inherent wholeness and undergoing a transformative process to remember this . . .is that this is how we discover we are more like others than not.” [68]. ST experience may elicit chills by confirming early, deep perceptual priors obscured by individuation in early development [29], of which we are reminded during peak life experiences.

## Limitations

These results may or may not be specific to characteristics of the participants. The sample is diverse and representative as regards the part of the world being sampled, but does not have equal representation of every subgroup within the sample, nor can it make overly strong claims regarding populations elsewhere in the country, in the world, or outside of WEIRD populations. The experimental context, while not strictly controlled by us as it is a survey study, exists within the same geographical context and is subject to similar constraints and assumptions. In terms of procedures, we are constrained by the explicit or latent idiosyncracies of the english language, and the conceptual framing used in the briefing. Regarding stimuli, here we encounter a bit of closed loop: the majority of the stimuli have been sourced by scraping social media (youtube) for videos that large numbers of users have rated as chills-inducing [44, 65]. Hence, the relationships and effects observed may, and likely do, reflect a matching between the largely WEIRD populations predominating on platforms like youtube, and the stimuli those populations denote as chills-inducing. While this speaks to the need for matching between subjective aesthetic stimuli and the populations for whom these aesthetics are familiar or compelling, it does not preclude that a similarly matched (using a platform dominated by east Asia, for example) set of stimuli would not elicit the same relationship between chills and characteristics of self-transcendence. We recently conducted a preregistered study using a subset of the stimuli used here, within a similarly diverse cohort in central Texas, and found nearly identical results [69]. Future studies should examine whether these relationships and effects hold in a progressively wider set of contexts, in other languages, and with a neutral or even misleading briefing. Indeed, the procedure outlined here and in [43, 44] is outlined in sufficient detail to permit replication and extension. We hope it will be carried out by other groups, in addition to our future efforts.

An additional limitation of this study is the reliance of self-reports, and a lack of neurophysiology or online (during stimulus presentation) reporting of phenomenology, limiting our ability to make causal claims about the order of psychological events. It is impossible from these results, for example, to conclusively state in what order participants noticed their chills response, the affective change, or the self-transcendent states (including whether these self-transcendent states were experienced first as emotions, or as perceptual states, etc.). However, the large cohorts do provide considerable validation of our stimuli, permitting us and other groups to perform on-site studies where we can reliably expect chills responses, allowing for these more fine-grained causal inquiries to be made.

## Conclusions

The results reported here support the use of stimuli selected for aesthetic chills (a marker of intense aesthetic experience) to replicably, and non-pharmacologically induce ST. In other words, stimuli selected for high likelihood and intensity of a pleasurable chills response are highly likely to also cause ST experiences, which are desirable from both a clinical and hedonic perspective. Given that chills can also be the result of cold, or horror, it seems likely that chills (and their intensity) denote experiences of high ST rather than causing them, though further study is needed. These effects approximate (though are likely less intense and long-lasting) those evoked by traditional, less accessible means such as psychedelics, peak life events, or advanced meditative practice [2, 5, 7, 8, 10, 13–15, 21–24, 37, 40, 70, 71]. However, even a low-level but replicable instance of ST may serve to aid and motivate novices in religious/meditative practices in cultivating the expertise to access ST at will. Given the numerous prosocial, meaning-making and well-being related outcomes attributed to ST, this work may have implications for tractably mitigating a wide variety of psychological and even societal issues. Future work should more rigorously examine the magnitude and longevity of effects of chills-based interventions, and whether chills-inducing media can be used in conjunction with other non-pharmacological methods to induce psychedelic-comparable, more clinically relevant (in magnitude and duration) states of ST. While ST appears generally salutogenic, there is evidence that persistent ST can, in some contexts, lead to deleterious effects [72]. By making ST more tractable to study we may better characterize the phenomenon and accompanying therapeutic considerations like dose-response curves and treatment personalization. Further work should also attempt more granular understandings and standardized, extensive measures of the phenomenology of ST, in which there is considerable reported variety [19]. Future research may benefit from facilitating the study of ST-inducing media in other locations and in clinical populations. We hope that efforts in the service of human flourishing will benefit from the procedures, stimuli, and data presented here.

## Supporting information

S1 Fig A. Histograms of traits across the full study cohort with overlaid density curves. Q-Q plots provide a reliable visual assessment of the data’s normality by comparing sample quantiles to theoretical quantiles of a normal distribution. Measures include MODTAS = Modified Tellegen Absorption Scale, KAMF = Kamamuta disposition scale, DPES = Dispositional Positive Emotion Scale, NEO-FFI-3 Five-Factor Inventory (Neuroticism, Extraversion, Openness, Agreeableness, Conscientiousness). B. Histograms of traits across the full study cohort with overlaid density curves. Q-Q plots provide a reliable visual assessment of the data’s normality by comparing sample quantiles to theoretical quantiles of a normal distribution. Measures include MODTAS = Modified Tellegen Absorption Scale, KAMF = Kamamuta disposition scale, DPES = Dispositional Positive Emotion Scale, NEO-FFI-3 Five-Factor Inventory (Neuroticism, Extraversion, Openness, Agreeableness, Conscientiousness).
(DOCX)

S2 Fig A. Histograms of outcome scores for the chills-only cohort, with overlaid density curves for each trait measure. Q-Q plots provide a reliable visual assessment of the data’s normality by comparing sample quantiles to theoretical quantiles of a normal distribution. Each graph represents a different measure, including chills intensity, Arousal delta (change post- > pre- stimulus), Valence delta, Mood delta, Ego-Dissolution (EDI), Connectedness (WCS), and Moral Elevation (SMES). B. Histograms of outcome scores for the chills-only cohort, with overlaid density curves for each trait measure. Q-Q plots provide a reliable visual assessment of the data’s normality by comparing sample quantiles to theoretical quantiles of a normal distribution. Each graph represents a different measure, including chills intensity, Arousal delta (change post- > pre- stimulus), Valence delta, Mood delta, Ego-Dissolution (EDI), Connectedness (WCS), and Moral Elevation (SMES).
(DOCX)

S1 Table Correlations between outcome measures before controlling for trait and demographic measures, stimulus, pre-stimulus affective state, and prior exposure. (DOCX)

S2 Table Exploratory factor analysis of all self-report questionnaire subscales. DPES = Disposition to Positive Emotion Scale, NEOFFI = five factor personality inventory, MODTAS = Modified Tellengen Absorption Scale, WCS = Watts Connectedness Scale, WCS = Watts Connectedness Scale.
(DOCX)
