# Supplementary material for: Self-transcendence accompanies aesthetic chills
Source: PLOS Ment Health. 2024 Oct 4;1(5):e0000125. doi: 10.1371/journal.pmen.0000125 (PMC12798208; doi:10.1371/journal.pmen.0000125)
Supplement: S1 Fig — A. Histograms of traits across the full study cohort with overlaid density curves. Q-Q plots provide a reliable visual assessment of the data’s normality by comparing sample quantiles to theoretical quantiles of a normal distribution. Measures include MODTAS = Modified Tellegen Absorption Scale, KAMF = Kamamuta disposition scale, DPES = Dispositional Positive Emotion Scale, NEO-FFI-3 Five-Factor Inventory (Neuroticism, Extraversion, Openness, Agreeableness, Conscientiousness). B. Histograms of traits across the full study cohort with overlaid density curves. Q-Q plots provide a reliable visual assessment of the data’s normality by comparing sample quantiles to theoretical quantiles of a normal distribution. Measures include MODTAS = Modified Tellegen Absorption Scale, KAMF = Kamamuta disposition scale, DPES = Dispositional Positive Emotion Scale, NEO-FFI-3 Five-Factor Inventory (Neuroticism, Extraversion, Openness, Agreeableness, Conscientiousness). (DOCX) [file pmen.0000125.s001.docx]

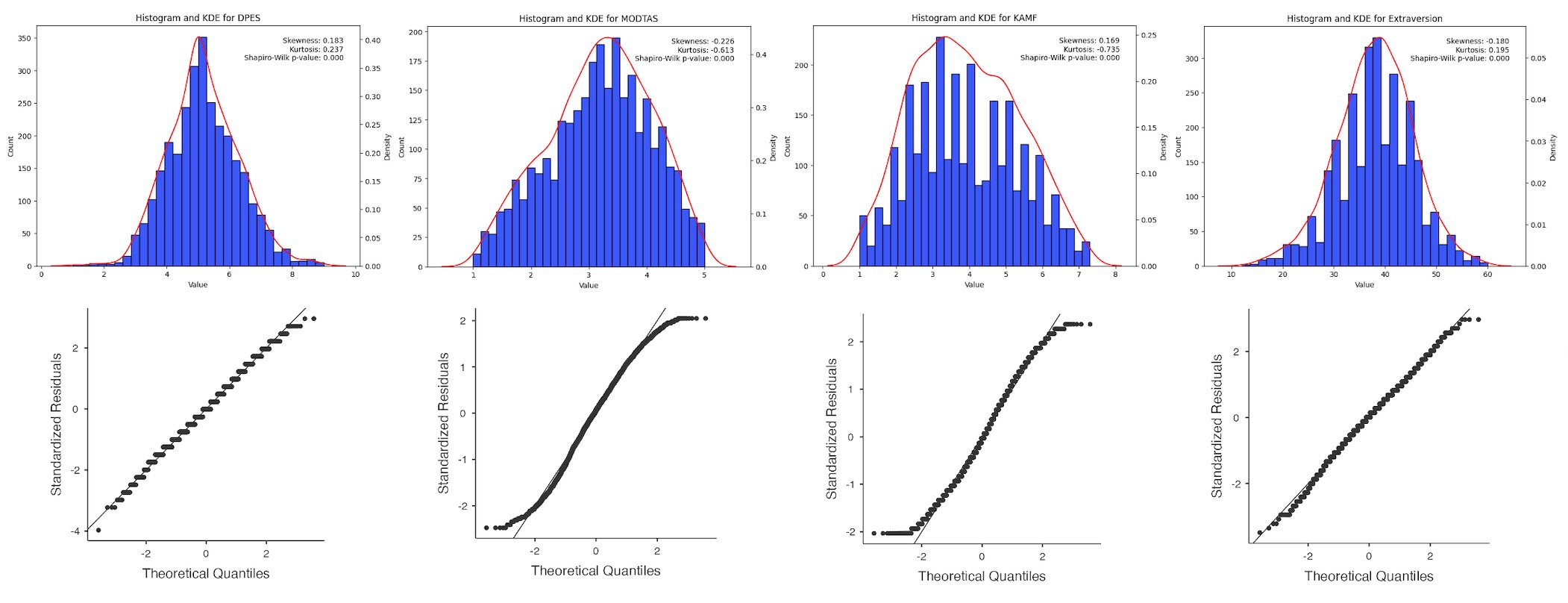


Supplementary Figure 1a.Histograms of traits across the full study cohort with overlaid density curves. Q-Q plots provide a reliable visual assessment of the data’s normality by comparing sample quantiles to theoretical quantiles of a normal distribution. Measures include MODTAS = Modified Tellegen Absorption Scale, KAMF = Kamamuta disposition scale, DPES = Dispositional Positive Emotion Scale, NEO-FFI-3 Five-Factor Inventory (Neuroticism, Extraversion, Openness, Agreeableness, Conscientiousness).


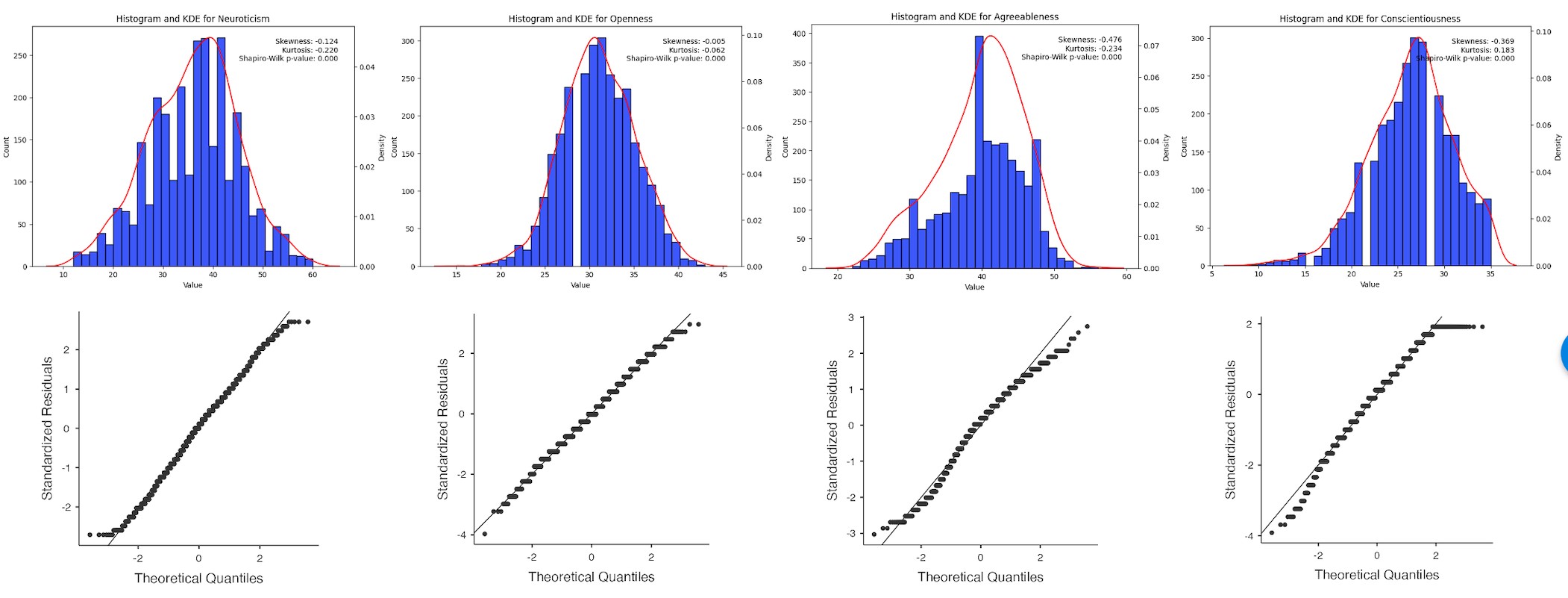


Supplementary Figure 1b.Histograms of traits across the full study cohort with overlaid density curves. Q-Q plots provide a reliable visual assessment of the data’s normality by comparing sample quantiles to theoretical quantiles of a normal distribution. Measures include MODTAS = Modified Tellegen Absorption Scale, KAMF = Kamamuta disposition scale, DPES = Dispositional Positive Emotion Scale, NEO-FFI-3 Five-Factor Inventory (Neuroticism, Extraversion, Openness, Agreeableness, Conscientiousness).
