# Supplementary material for: Self-transcendence accompanies aesthetic chills
Source: PLOS Ment Health. 2024 Oct 4;1(5):e0000125. doi: 10.1371/journal.pmen.0000125 (PMC12798208; doi:10.1371/journal.pmen.0000125)
Supplement: S2 Fig — A. Histograms of outcome scores for the chills-only cohort, with overlaid density curves for each trait measure. Q-Q plots provide a reliable visual assessment of the data’s normality by comparing sample quantiles to theoretical quantiles of a normal distribution. Each graph represents a different measure, including chills intensity, Arousal delta (change post- > pre- stimulus), Valence delta, Mood delta, Ego-Dissolution (EDI), Connectedness (WCS), and Moral Elevation (SMES). B. Histograms of outcome scores for the chills-only cohort, with overlaid density curves for each trait measure. Q-Q plots provide a reliable visual assessment of the data’s normality by comparing sample quantiles to theoretical quantiles of a normal distribution. Each graph represents a different measure, including chills intensity, Arousal delta (change post- > pre- stimulus), Valence delta, Mood delta, Ego-Dissolution (EDI), Connectedness (WCS), and Moral Elevation (SMES). (DOCX) [file pmen.0000125.s002.docx]

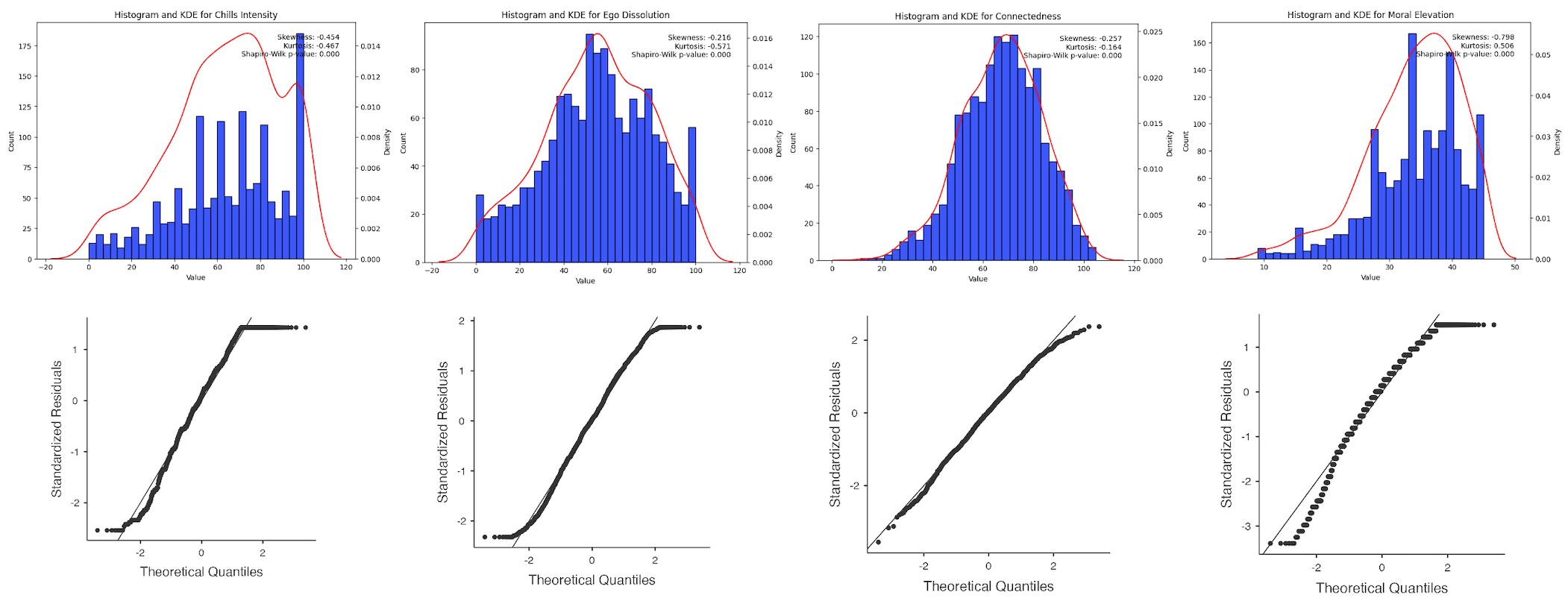


Supplementary Figure 2a.Histograms of outcome scores for the chills-only cohort, with overlaid density curves for each trait measure. Q-Q plots provide a reliable visual assessment of the data’s normality by comparing sample quantiles to theoretical quantiles of a normal distribution. Each graph represents a different measure, including chills intensity, Arousal delta (change post- > pre- stimulus), Valence delta, Mood delta, Ego-Dissolution (EDI), Connectedness (WCS), and Moral Elevation (SMES)


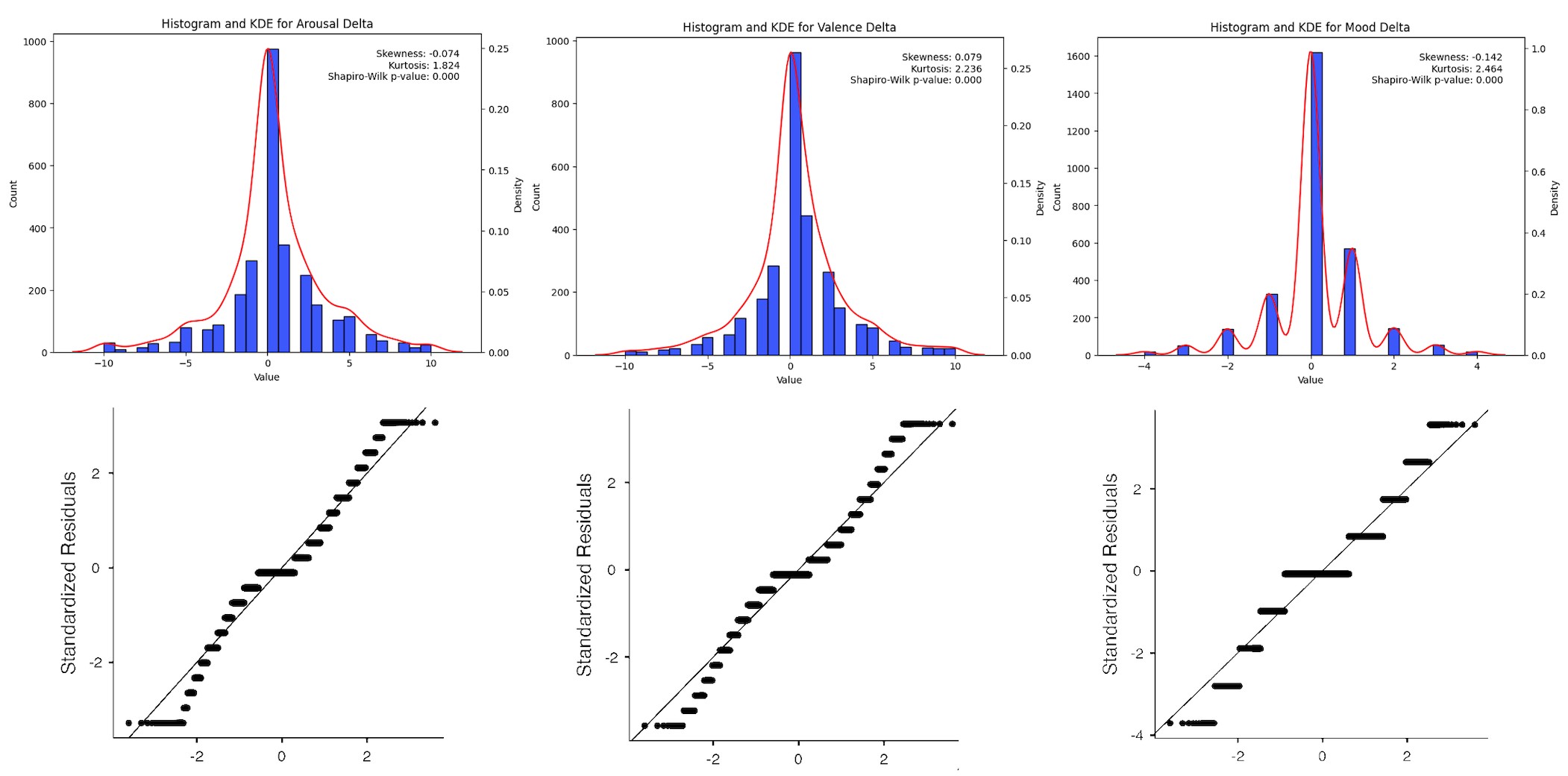


Supplementary Figure 2b.Histograms of outcome scores for the chills-only cohort, with overlaid density curves for each trait measure. Q-Q plots provide a reliable visual assessment of the data’s normality by comparing sample quantiles to theoretical quantiles of a normal distribution. Each graph represents a different measure, including chills intensity, Arousal delta (change post- > pre- stimulus), Valence delta, Mood delta, Ego-Dissolution (EDI), Connectedness (WCS), and Moral Elevation (SMES)
