# Supplementary material for: Self-transcendence accompanies aesthetic chills
Source: PLOS Ment Health. 2024 Oct 4;1(5):e0000125. doi: 10.1371/journal.pmen.0000125 (PMC12798208; doi:10.1371/journal.pmen.0000125)
Supplement: S1 Table — (DOCX) [file pmen.0000125.s003.docx]

Table 1: Correlations between outcome measures before controlling for trait and demographic measures, stimulus, pre-stimulus affective state, and prior exposure.

| Variable | Chills Ego Connectedness Moral ∆Arousal ∆Valence | | | | | |
| --- | --- | --- | --- | --- | --- | --- |
| Intensity Dissolution | | | | Elevation | |  |
| Chills Intensity . | | . | . | . | . | . |
| Ego Dissolution .516* | | . | . | . | . | . |
| Connectedness .480* | | .511* | . | . | . | . |
| Moral Elevation .506* | | .550* | .643* | . | . | . |
| ∆Arousal .010 | | -.007 | -.026 | -.013 | . | . |
| ∆Valence .015 | | -.011 | .078* | .038 | .016 | . |
| ∆Mood -.002 | | -.009 | .031 | -.007 | -.099* | .264* |
