# Supplementary material for: Self-transcendence accompanies aesthetic chills
Source: PLOS Ment Health. 2024 Oct 4;1(5):e0000125. doi: 10.1371/journal.pmen.0000125 (PMC12798208; doi:10.1371/journal.pmen.0000125)
Supplement: S2 Table — DPES = Disposition to Positive Emotion Scale, NEOFFI = five factor personality inventory, MODTAS = Modified Tellengen Absorption Scale, WCS = Watts Connectedness Scale, WCS = Watts Connectedness Scale. (DOCX) [file pmen.0000125.s004.docx]

Table 2: Exploratory Factor Analysis of all self-report questionnaire subscales.

Factor

Factor Loadings 1 2 3 4 5 6 7 Uniqueness

| DPES \| Compassion | . | . | .518 . | | . | . | . | .744 |
| --- | --- | --- | --- | --- | --- | --- | --- | --- |
| DPES \| Awe | . | . | .687 . | | . | . | . | .484 |
| DPES \| Joy | . | . | .7 . | | . | . | . | .446 |
| DPES \| Love | . | . | .658 . | | . | . | . | .509 |
| DPES \| Pride | . | . | .632 . | | . | . | . | .536 |
| DPES Amusement | . | . | .568 . | | . | . | . | .606 |
| NEOFFI-Neuroticism \| Negative Affect | . | . | . | . | . | .484 .453 | | .522 |
| NEOFFI-Neuroticism \| Self-Reproach | . | . | . | . | . | .822 . | | .138 |
| NEOFFI-Extraversion \| Positive Affect | . | . | . | .736 | . | . | . | .355 |
| NEOFFI-Extraversion \| Sociability | . | . | . | .757 | . | . | . | .371 |
| NEOFFI-Extraversion \| Activity | . | . | . | .389 | . | . | -.369 .309 | |
| NEOFFI-Openness \| Aesthetic Interests | .398 . | | . | . | . | . | .345 | .609 |
| NEOFFI-Openness \| Intellectual Interests | .36 . | | . | . | . | . | .345 | .678 |
| NEOFFI-Openness \| Unconventionality | . . | | . | . | . | -.47 | . | .458 |
| NEOFFI-Agreeableness \| Nonantagonism | . . | | . | . | . | . | .696 | .288 |
| NEOFFI-Agreeableness \| Prosociality | . . | | . | .301 | . | . | . | .732 |
| NEOFFI-Conscientiousness \| Goal Striving | . . | | . | . | .845 . | | . | .22 |
| NEOFFI-Conscientiousness \| Dependability . . | | | . | . | .687 . | | . | .45 |
| MODTAS \| synesthesia | .828 . | | . | . | . . | | . | .239 |
| MODTAS \| ASC | .816 . | | . | . | . . | | . | .208 |
| MODTAS \| aesthetics | .836 . | | . | . | . . | | . | .264 |
| MODTAS \| imagination | .897 . | | . | . | . . | | . | .173 |
| MODTAS \| ESP | .801 . | | . | . | . . | | . | .385 |
| WCS \| Self | . .9 | | . | . | . . | | . | .197 |
| WCS \| Others | . . | | . | . | . . | | . | .785 |
| WCS \| World | . .97 | | . | . | . . | | . | .083 |
| Ego Dissolution | . .881 . | | | . | . . | | . | .202 |
| Moral Elevation | . .824 . | | | . | . . | | . | .191 |

DPES = Disposition to Positive Emotion Scale, NEOFFI = five factor personality inventory, MODTAS = Modified Tellengen Absorption Scale, WCS = Watts Connectedness Scale, WCS = Watts Connectedness Scale
